# Using nominal group technique among resident physicians to identify key attributes of a burnout prevention program

**DOI:** 10.1371/journal.pone.0264921

**Published:** 2022-03-18

**Authors:** Vicki Nelson, Alex Dubov, Kelly Morton, Liana Fraenkel

**Affiliations:** 1 School of Religion, Loma Linda University Health, Loma Linda, California, United States of America; 2 School of Behavioral Health, Loma Linda University Health, Loma Linda, California, United States of America; 3 Research Department, Family Medicine, School of Medicine, Loma Linda University Health, Redlands, California, United States of America; 4 Rheumatology Department, Berkshire Medical Center, Pittsfield, Massachusetts, United States of America; 5 Patient Centered Population Health Research, Berkshire Health Systems, Pittsfield, Massachusetts, United States of America; 6 Yale University School of Medicine, New Haven, Connecticut, United States of America; Universitat de Valencia, SPAIN

## Abstract

**Purpose:**

To identify preferred burnout interventions within a resident physician population, utilizing the Nominal Group Technique. The results will be used to design a discrete choice experiment study to inform the development of resident burnout prevention programs.

**Methods:**

Three resident focus groups met (10–14 participants/group) to prioritize a list of 23 factors for burnout prevention programs. The Nominal Group Technique consisted of three steps: an individual, confidential ranking of the 23 factors by importance from 1 to 23, a group discussion of each attribute, including a group review of the rankings, and an opportunity to alter the original ranking across participants.

**Results:**

The total number of residents (36) were a representative sample of specialty, year of residency, and sex. There was strong agreement about the most highly rated attributes which grouped naturally into themes of autonomy, meaning, competency and relatedness. There was also disagreement on several of the attributes that is likely due to the differences in residency specialty and subsequently rotation requirements.

**Conclusion:**

This study identified the need to address multiple organizational factors that may lead to physician burnout. There is a clear need for complex interventions that target systemic and program level factors rather than focus on individual interventions. These results may help residency program directors understand the specific attributes of a burnout prevention program valued by residents. Aligning burnout interventions with resident preferences could improve the efficacy of burnout prevention programs by improving adoption of, and satisfaction with, these programs. Physician burnout is a work-related syndrome characterized by emotional exhaustion, depersonalization, and a sense of reduced personal accomplishment [1]. Burnout is present in epidemic proportions and was estimated to occur in over 50 percent of practicing physicians and in up to 89 percent of resident physicians pre-COVID 19. The burnout epidemic is growing; a recent national survey of US physicians reported an 8.9 percent increase in burnout between 2011 and 2014 [2]. Rates of physician burnout have also increased [3] during the COVID-19 pandemic with a new classification of “pandemic burnout” experienced by over 52 percent of healthcare workers as early as June of 2020 [4]. Physician burnout can lead to depression, suicidal ideation, and relationship problems that may progress to substance abuse, increased interpersonal conflicts, broken relationships, low quality of life, major depression, and suicide [5–7]. The estimated rate of physician suicide is 300–400 annually [8–10].

## Introduction

It often takes a tragedy like the one suffered at a New York City hospital where two first-year residents died by suicide within four days, to draw attention to the seriousness of the problem [11]. A 2017 study of medical residents and causes of death found that suicidal ideation increased 370 percent over the first three months of the first year of training [12]. Burnout also negatively affects physicians’ friends and family, patient care and safety, as well as healthcare organizations and systems. In a clinical setting, evidence demonstrates that burnout results in prescription errors, and reduces the quality of medical services potentially affecting inter-professional relationships [5–7, 13]. According to recent estimates, the burnout epidemic costs the healthcare system approximately $4.6 billion a year [13].

Existing research on burnout prevention among residents focuses primarily on physician-directed interventions, such as building personal resiliency [14] and practicing mindfulness-based stress reduction [15] A recent meta-analysis of 20 controlled studies evaluating over 1500 physicians found that these interventions are associated with insignificant benefits and suggested the focus should be on the adoption of organization-directed approaches [16]. Thus, there is an urgent need to design and deliver effective interventions aimed to reduce burnout that will result in high uptake by medical residents that may extend to other physician groups. Understanding how residents value various aspects of burnout interventions is vital to both the design and evaluation of burnout prevention programs [17]. Aligning burnout interventions with residents’ preferences could improve the effectiveness of burnout prevention programs by improving adoption of, and satisfaction with, these programs.

Discrete choice experiment (DCE) research can be used to understand how stakeholders value specific components of possible programs or interventions. For example, this approach was used to model the physician preferences regarding a pay-for performance incentive program, [18] adherence promoting programs, [19] or residency choices of medical students [20]. Programs developed based on stakeholders’ preferences result in improved adoption and satisfaction with the program. Existing burnout prevention programs are rarely informed by medical residents and are consequently often underutilized. We seek to design a burnout prevention program based on the preferences of medical residents using a DCE study. The first step in designing a DCE study is to identify which attributes to include in the study design. The purpose of this focus group study is to identify the attributes to include in a future DCE study using the Nominal Group Technique (NGT).

## Methods

Three resident focus groups (consisting of 10–14 participants/session) were conducted in July and August 2020, in three medical centers in California to prioritize resident preferences for burnout intervention attributes. We purposely chose programs based at diverse healthcare organizations. The first program was based at a university with 900 resident physicians across all medical specialties. Residents rotated at both the university hospital and the affiliated Veterans Affairs Medical Center. The second program was based at a large (476 beds) not-for-profit medical center with family medicine, internal medicine, and emergency medicine residency programs (over 100 residents in total). The third program was based at a community teaching hospital with 14 residents enrolled in a family medicine residency program.

All residents were emailed an invitation to participate in a focus group at a predetermined time. Anyone who presented the day of the focus group could participate. Due to the COVID-19 pandemic, two focus groups had to meet via Zoom, while one group met in person. Participants were considered eligible for inclusion in the study if they were currently part of a residency program at one of these three centers. They were purposely selected to represent the full program spectrum of age, sex, specialty, type of hospital, and year of residency.

The moderator had no reporting relationships with residents or faculty in any participating program, and a neutral note-taker was present during each focus group. Participants provided oral informed consent before discussions began. Focus groups were audio-recorded and transcribed; all identifiers were removed before analysis. After each focus group, participants were provided with information on available resources for addressing burnout. The Loma Linda University institutional review board approved the study as expedited.

### Study design

NGT was selected as a strategy for identifying relevant attributes and relative ranking of importance. NGT is a qualitative exploratory research method often used in situations that require complex problem solving, decision-making, priority-setting, and consensus reaching [21]. It is defined as “a structured method for group brainstorming that encourages contributions from everyone and facilitates quick agreement on the relative importance of issues, problems, or solutions” [21–23]. Participants present their favorite ideas, and the suggestions are then discussed and prioritized by the entire group. In this way NGT combines the importance ratings of individual group members into the final weighted priorities of the group.

NGT combines quantitative and qualitative data collection in a group setting while avoiding the issues of group dynamics present in other group methods such as Delphi methods or brainstorming. Additional advantages of NGT over other group methods include reduced researcher bias due to the structured nature of the process, expedient achievement of data saturation, and its ability to simultaneously address problem identification, development of solutions, and establishing priorities for action. This method can be implemented within a narrow timeframe, and it is easily understood by participants. Responses are weighted by participants, and the process is transparent, inclusive and can be easily replicated. NGT promotes even participation and facilitates representation of the implicit views of the group [24–26].

### Procedures

**Fig 1:**
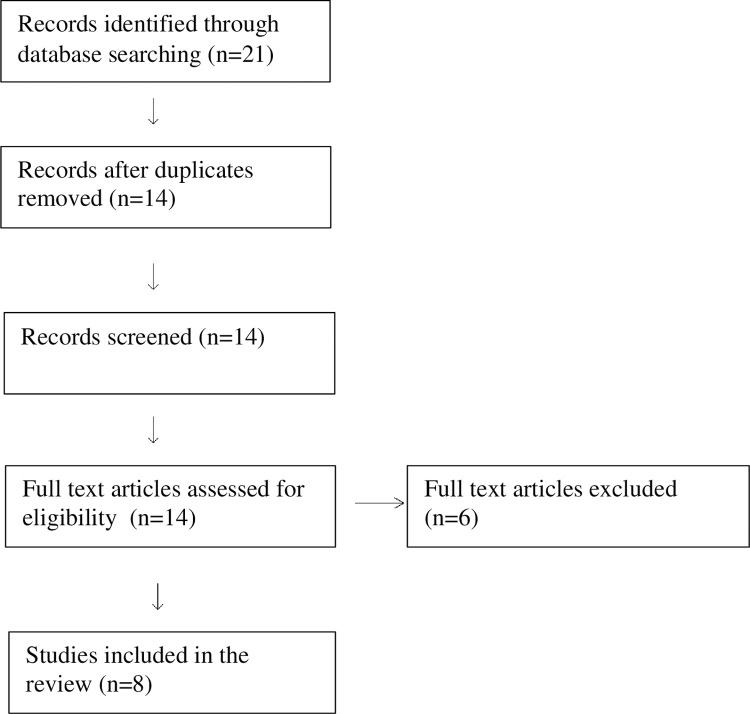
Flow chart.

We conducted a literature search to identify systematic reviews of interventions to reduce burnout in groups of physicians (medical residents, interns, practicing physicians). Both individual and organization directed interventions were included. We restricted our review of existing studies to a time limit of 10 years and found eight systematic reviews [16, 17, 27–32]. Table 1 lists key characteristics of the systematic reviews, and a flow chart is included as Fig 1.

**Table 1:** Characteristics of systematic reviews.

Authors	Type of review (time range)	# of studies	Country	Research aim or question	Research design	Measurements
Panagioti M, et.al	Systematic review (2005–2016)	19	US	To evaluate the effectiveness of interventions to reduce physician burnout and investigating relationship between different types of interventions, physician characteristics, and healthcare setting characteristics with improved effect.	RCT Controlled before-after studies	MBI
Busrireddy, et.al	Systematic review (2004–2015 and one article 1991)	19	US	To explore the efficacy of interventions in reducing resident burnout.	RCT Cohort studies	MBI
Clough, et.al	Systematic review (2007–2018)	23	UK	to review and evaluate evidence on psychosocial interventions aimed at reducing occupational stress and burnout among medical doctors.	RCT Pre-post intervention Quasi-experimental	MBI
Dechant, et.al	Systematic review (2007–2018)	50	US	To assess the impact of organization-directed workplace interventions on physician burnout, including stress or job satisfaction in all settings	RCT Cross-sectional Pre-post intervention Quasi-experimental Prospective cohort	MBI, PJSS, ESS, OWL, MEMOS, OLM, PWS, NSCWS, MHCS, ACPIMPS, IM
Kalani, et. al	Systematic review (2008–2016)	4	Iran	To systematically review systematic review studies of interventions for physician burnout to evaluate and summarize their results.	Review studies Systematic reviews	
Perski, et. al	Systematic review (2006–2011)	8	UK	To assess the effect of tertiary interventions for individuals with clinical burnout	RCT Cluster RCT Non-randomized controlled trial	RTW
West, et. al	Systematic review and meta-analysis (2003–2015 and one article 1998)	52	UK, US, China	To understand the quality and outcomes of the interventions to prevent and reduce physician burnout.	RCT Cohort studies	MBI, EES, DS
Walsh, et. al	Systematic review (2006–2016 and one article 1991)	14	Switzerland	To identify interventions to prevent and/or reduce burnout among medical students and residents.	Single group pre-post studies RCT Non-randomized two group studies	MBI

Maslow’s Burnout Inventory (MBI), Physician Job Satisfaction Scale (PJSS), Epworth Sleepiness Scale (ESS), Office and Work Life Measures (OWL), Minimizing Error, Maximizing Outcome Scale (MEMOS), Office and Lifework Measures (OLM), Physician Worklife Study (PWS), National Study of Changing Workforce Scale (NSCWS), Massachusetts eHealth Collaborative Survey (MHCS), American College of Physicians/American Society for Internal Medicine physician satisfaction (ACPIMPS), Independent measures (IM), Return to Work (RTW), Emotional Exhaustion Score (EES), Depersonalization Score (DS)

### Flow Chart

Twenty-three potential important attributes of intervention programs were established from this literature review. Table 2 lists the attributes numbered from one to twenty-three.

**Table 2:** List of potentially important attributes/interventions.

1.	Mindfulness and self-care training
2.	Limiting duration of shifts
3.	Enforcing sensible patient censuses
4.	One-on-one counseling geared to improve wellness
5.	Flexible scheduling allowing for time off work
6.	Increased direct patient contact, protected time with patients
7.	Group meetings with physician-leaders, physician communities, peer-discussion groups
8.	Brief evidence-based stress reduction interventions during shifts
9.	Periods of protected sleep time during shift
10.	Incentivizing physical exercise programs/gym attendance
11.	Communication skills training (e.g., dealing with difficult patients)
12.	Stress management training (focus on distress, emotional exhaustion etc.)
13.	Nonphysician staff support to offload clerical burdens
14.	Time banking intervention, allowing time to focus on professional development/meaningful activities
15.	Hold a forum for residents to voice concerns and influence change
16.	Improvement in physical work environment–better sleep rooms, creating shared spaces/break rooms
17.	Emphasize learning over service–protect formal educational time, minimal responding clinical duties during educational conferences
18.	Schedule residents to work together on consistent teams
19.	Encourage faculty to provide more frequent positive feedback
20.	Spiritual nurturing and care
21.	Creative art therapy
22.	Residency-wide social events
23.	Develop formal, accessible mechanisms for reporting and investigating sexual harassment and racial discrimination

The NGT process consisted of three steps. After a welcome from the facilitator, participants were asked to introduce themselves, including specialty and year of residency. The facilitator then described the purpose of the study, the process, and the survey list of potential attributes with a description of each attribute. These attributes were displayed in Google Sheets for all participants to view in real-time. Participants were then asked to complete the survey, ranking each potential attribute from 1–23 with one being the most important and twenty-three being the least. Ranking occurred anonymously using Google Sheets. Each participant was assigned an individual sheet displaying the list of attributes in one column and the drop-down list of ranking values (1–23) in the adjacent column. Participants ranked each attribute, assigning only one number per attribute. The aggregate ranking of each attribute was instantly updated on a separate summary sheet available to the group facilitator. The summary sheet displayed individual anonymous rankings of each group member (e.g., column B) together with the list of attributes sorted according to the rank order at the group level.

During a second step (discussion and sharing ideas), a group discussion on each of the attributes was held and included a group review of the aggregate score of the initial ranking, displayed in the summary sheet and presented to participants. This discussion reviewed if the group endorsed the highest-ranking attributes and contextualized responses and the rank-ordering. After the three group discussions, the rank order of the attributes was tallied. Finally, residents had an opportunity to reconsider their initial ranking of the attributes based on the group discussion. They were not pressured to alter their ranking nor to achieve consensus. The facilitator endeavored to ensure that all participants were given the opportunity to contribute. None of the participants opted to change their ranking of attributes following discussion.

## Results

A total of 36 residents participated across the three sites with 10, 12, and 14 participants per focus group. As seen in Table 3, the residents were a representative sample of specialty, year of residency, sex, and program location. Internal Medicine and Family Medicine were the specialties represented by the greatest number of participants (n = 16 and n = 15) followed by a small number of residents from Anesthesia, Surgery, and Emergency Medicine. The groups were almost equally divided by sex and year of residency.

**Table 3:** Participants’ characteristics.

Total Participants	36	(N = 36)
	Women	(n = 19, 53%)
	Men	(n = 17, 47%)
**Facility**		
	University Hospital	(n = 10, 28%)
	Medical Center	(n = 12, 33%)
	Community Hospital	(n = 14, 39%)
**Year of Residency**		
	PGY1	(n = 9, 25%)
	PGY2	(n = 11, 30%)
	PGY3	(n = 13, 36%)
	PGY4 and over	(n = 3, 9%)
**Specialty**		
	Internal Medicine	(n = 16, 44%)
	Family Medicine	(n = 15, 42%)
	Anesthesia	(n = 2, 5.5%)
	Surgery	(n = 2, 5.5%)
	Emergency Medicine	(n = 1, 3%)

“Using the direct rating method,” we calculated mean importance scores and standard deviations for each program attribute. The mean importance score was calculated by dividing the total points awarded per attribute by the total number of residents participating in the focus groups. The mean scores show the group aggregate rank, whereas standard deviation show the spread, i.e., disagreement of the group’s responses around that result. Based on the mean importance score and calculated standard deviations, we made the ranking of attributes from most (lowest mean) to least (highest mean) important (Table 4). Similar to other studies using nominal group technique to identify attributes for inclusion in discrete choice experiments, we plan to include the top 10 most important attributes in our follow-up study. Consequently, only top 10 ranked attributes are highlighted in the discussion [33, 34]. The highest agreement among participants with the resulting smallest standard deviation was from attribute 6 –***emphasize learning over service–***with participants agreeing that it is a useful intervention (4.74 standard deviation). Next highest agreement, attribute 20 –***communication skills training***–with participants agreeing that it is not a useful intervention (4.87). The highest prioritized intervention was attribute 1 –***flexible scheduling***–with participants agreeing that this is the most wanted intervention, though with a higher standard deviation (4.91). The highest disagreement among participants with the resulting highest standard deviation was attribute 21, ***spiritual nurturing and care*** (7.51), followed by attribute 10, ***mindfulness and self-care training*** (7.40) and attribute 5, ***enforcing sensible patient censuses*** (6.71). This disagreement can be attributed to the differences in residency programs and types of hospital settings. One setting was a faith-based organization and some residents spent more time in outpatient versus inpatient settings that require the consideration of a patient census limit for trainees.

**Table 4:** Rank order of attributes.

Rank	INTERVENTION	Total points	Mean Imp Sco	Std Dev
1.	Flexible scheduling allowing for time off work	201	6.09	4.91
2.	Limiting duration of shifts	218	6.61	5.80
3.	Incentivizing physical exercise programs/gym attendance	284	8.61	5.47
4.	Periods of protected sleep time during shifts	289	8.76	6.44
5.	Enforcing sensible patient censuses	307	9.30	6.71
6.	Emphasize learning over service–protect formal educational time, minimal responding clinical duties during educational conferences	316	9.58	4.74
7.	Non-physician staff support to offload clerical burdens	323	9.79	6.37
8.	Hold a forum for residents to voice concerns and influence change	346	10.48	6.10
9.	Residency-wide social events	352	10.67	6.32
10.	Mindfulness and self-care training	365	11.06	7.40
11.	Stress management training (focus on distress, emotional exhaustion)	404	12.24	6.47
12.	Encourage faculty to provide more frequent positive feedback	410	12.42	5.83
13.	Time banking intervention, allowing time to focus on professional development/meaningful activities	413	12.52	6.06
14.	Improvement in physical work environment–better sleep rooms, creating shared spaces/break rooms	424	12.85	5.91
15.	One-on-one counseling geared to improve wellness	459	13.91	5.57
16.	Increased direct patient contact, protected time with patients	462	14.00	5.81
17.	Group meetings with physician-leaders, physician communities, peer-discussion groups	484	14.67	5.33
18.	Brief evidence-based stress reduction interventions during shifts	490	14.85	5.46
19.	Schedule residents to work together on consistent teams	495	15.00	6.38
20.	Communication skills training (e.g., dealing with difficult patients)	503	15.24	4.88
21.	Spiritual nurturing and care	507	15.36	7.52
22.	Develop formal, accessible mechanisms for reporting and investigating sexual harassment and racial discrimination	518	15.70	6.39
23.	Creative art therapy	549	16.64	6.42

We review the top 10 priority interventions and provide participants’ quotes to help contextualize the ratings in the following sections.

### Flexible schedule allowing for time off work

This attribute was identified as the essential aspect of the burnout prevention programs. In all three focus groups, residents prioritized time for personal and professional needs as the most critical aspect of burnout prevention. Residents talked about their time constraints preventing them from practicing self-care, mindfulness, or meaningful learning. Time off work and flexibility in scheduling was discussed as a baseline for any other wellness intervention. When on-call schedules do not allow residents to meet basic needs such as sleep and self-care, any additional intervention to prevent burnout is meaningless.

“*Scheduling is so important*. *This is a change that doesn’t take much work to implement and will have a real*, *immediate impact*. *It is no stretch of the imagination to think working fewer days in a row could mean a better quality of life*. *More rest between shifts means a better quality of life*. *More time-off requests granted means more of us are happier in the long-term*. *This is the most immediate and tangible solution instead of stress management or meditation promoted by some programs*.”

### Limiting the duration of shift

Residents discussed the importance of fatigue as a precursor to burnout. They talked about how long hours, heavy workloads, and constant pressure are emotionally taxing. Participants emphasized the ability to spend time away from work to reenergize, engage in activities that can prevent burnout, and maintain a life outside of work.

“*Having shorter shifts means I have time either before or after my shift to spend time with my family*, *work out*, *cook meals*, *and be a normal person outside of the hospital*.*”*

*“Having a shorter shift structure would let me recover sooner after night shifts*. *You are not completely wiped out at the end of your shift*.”

### Physical exercise

Residents described the protective mechanisms of physical fitness and exercise. Participants spoke about the benefits of exercise not only for physical, but also for their mental health and well-being. There was a discrepancy in ranking this intervention. University hospital residents ranked it lower than the other two programs, possibly due to their easy access to a state-of-the-art recreation and wellness center.

“*Exercise is the best way for me to sweat off stress and anxiety*. *I keep talking to my patients about the importance of physical activity*, *yet my schedule is so hectic that I cannot find enough time to exercise regularly*. *It takes about 20 minutes to get to the gym*, *and I feel like our minutes are precious*. *I am struggling to integrate exercise into my routine*.*”*

### Protected sleep time

Despite the work hours restrictions, it remains challenging to get enough sleep while in residency. Residents linked sleep deprivation to burnout and discussed this issue as requiring system change rather than individual attention. They mentioned difficulties in noticing and addressing their sleep deprivation, exacerbated by an “I can handle it” mentality.

*“Once I ended up being on call for six days in a row. Near the end, I was so tired that I stopped smiling. I was so tired of running on no sleep. During that time, we were getting late-night calls from the ER, but you are expected to see patients during the day as well. So, you are running on no sleep. At the end of the sixth day, I developed a fever and had to call in sick.”*

### Sensible patient census

Residents articulated the distinction between hours worked per shift and the number of admissions during the shift. Because of work hour restrictions by accreditation policies, [35] programs may only focus on hours rather than patient numbers. While their programs seemingly understand the connection between burnout and workload, residents perceived their program directors being more concerned with burnout resulting from hours worked per shift rather than the intensity of work completed during those hours. Enforcing sensible patient census to control workload was a priority for the university hospital and medical center, but less critical for the community hospital residency.

*“I can have several long shifts scheduled back-to-back but uneventful and still feel fine. Sometimes my shifts are more staggered and seem more manageable, but I end up caring for a lot more patients or dealing with many end-of-life situations and feel much more run down. It is not only about how many hours I spent in the hospital, but also what happens during those hours that matters for burnout.”*

### Protected learning time

Meaningful learning experiences are essential during residency. Participants emphasized the fact that residency is an educational experience. They work long hours for minimal pay in exchange for the benefits of clinical education. For residents to receive the most benefit from their training, programs need to prioritize their learning by securing uninterrupted educational time. This protected time would show residents that hospitals value their personal growth and training over their service.

*“It is refreshing to have an opportunity to invest in your education and improvement as a physician. At least in part, my burnout experience was connected to a feeling of not being prepared to face some of the more challenging clinical situations. One solution is taking some time away from chaos, turning off your pager, and having a meaningful learning experience. I listen better, ask more questions, and learn more when I can have no responding duties during educational conferences.”*

### Non-physician staff to offload EHR imposed stress

According to a recent study, the average physician spends 28 hours on clinical documentation during nights and weekends each month [21]. Residents in this study reported a high level of stress from Electronic Health Record (EHR) systems and dissatisfaction from reduced direct face time with patients. One resident commented:

*“At times, I feel like EHR robs me of everything that gives me meaning in my profession–it makes me less efficient, less able to spend time with my patients. It is very disruptive to the flow of my work.”*

### Voice for residents to influence change

Residents identified programs’ unresponsiveness to residents’ feedback as one of the factors contributing to burnout. Residents perceived themselves as being at the bottom of the hospital hierarchy and rarely having control over their hours, schedule, and work environment. Having a sense of control and being able to influence change can moderate the intensity of workload and burnout. Residents prioritized the ability to have a forum where they can voice concerns and see them addressed.

*“Giving residents a voice as much as possible is a great way to sustain our motivation and prevent apathy. Let us tell you what works and what interventions are effective (or not). We are the ones on the frontlines.”*

### Social connections

Participants discussed multiple ways in which residency programs and healthcare practice can lead to a sense of isolation and loneliness, resulting in burnout. They talked about many hours spent in front of their computers working on medical records, high case workload, and online learning, leading to reduced face-to-face interactions. They highlighted the connection between social support and reduced burnout.

*“One of my priorities as of late is to invest in relationships. Residency can be lonely and isolated. This sense of isolation can lead to burnout. I believe we need more personal, human interaction beyond quick, professional exchanges. We need opportunities to talk with each other about our work and lives. We need to vent about bad stuff, to talk about meaningful experiences at work, to share tips about surviving this place without losing our sanity.”*

### Mindfulness

Some residents found mindfulness useful for combating stress and burnout. They talked about the importance of self-awareness and positive self-reinforcement to reduce their emotional response to stressors.

*“I’ve been focusing on self-care and fostering my sense of connection, not only to family and friends, but to myself. I am figuring out what really inspired me, what really motivated me. This practice of mindfulness can be very cathartic or Zen.”*

However, there was a high level of disagreement between the usefulness of mindfulness and stress-management classes to reduce burnout. Residents at the community hospital ranked this intervention remarkably high, while residents at the university hospital ranked it exceptionally low.

*“Our program focuses a lot on this. Pretty much every retreat we go through, that is the one thing they do. And I am so glad to see the agreement that this is not helpful.”*

## Discussion

Although burnout during residency training has been widely acknowledged, there is less consensus regarding ways to address this problem [16, 17]. Little is known about which interventions among this variety of burnout prevention strategies residents value the most. This study provides an overview of the interventions residents value most to combat burnout, and what makes those interventions valuable. Our findings offer insight that may help focus future prevention efforts.

The study results suggest that residents prioritize interventions targeting systemic and program-level factors contributing to burnout over the individual interventions. This finding is in line with several recently published studies [36–38]. Participants connected burnout experience to their working conditions and characteristics of their programs rather than their failure to maintain a proper work-life balance or inability to manage stress. A variety of factors contribute to residents’ burnout: reduced sense of control over their schedule, their workload, time spent with patients, and their learning; reduced sense of belonging and social connectedness; reduced time for self-care and patient care; reduced sense of meaning and purpose. As a result, participants considered systems factors the most critical target for interventions, rather than individual strategies such as stress management. They prioritized flexible schedules and protected learning time to increase ***control*** over their work and learning environment and have a voice and ability to influence change. Residents wanted their programs to cultivate ***meaning*** in residency training by bringing them back to patient care (e.g., relieving the EHR burden). They talked about addressing the reasons why they are pulled away from the bedside, such as high patient census and high volume of administrative tasks. Participants referred to ***time*** as the priority for interventions to reduce burnout–limiting durations of shifts to allow for self-care and protecting sleep time. Finally, they mentioned the importance of social connectivity and practicing within a work environment that fosters a sense of ***belonging***.

Residents prioritized several individual-level interventions such as physical exercise programs and mindfulness training. However, these interventions were on the list of the attributes with the highest disagreement among participants. Residents of one program were dissatisfied with the sole focus of their program administration on boosting physicians’ resilience as the best strategy for dealing with burnout. It is also worth mentioning the contextual differences between programs that factored into residents’ preferences. While a physical exercise program was highly prioritized by medical center and community hospital residents, it was scored lower by the university hospital residents who had easy access to an on-site, state-of-the-art wellness center that remained open in the pandemic with classes outdoors. Stress management and mindfulness training were a high priority for community hospital residents, and a low priority for medical center and university hospital residents who had this incorporated by their programs and realized it was not helpful.

The results of our study support the need for interventions to address multiple organizational factors leading to burnout as opposed to addressing a single issue such as workload or shift patterns. There is a need for complex interventions that will target several systemic and program level factors [39, 40] while including individual-level interventions when indicated. Organizational psychology suggests several factors that may support intrinsic motivation of residents: autonomy, meaning, competence, and relatedness. As our study shows, these factors tend to be reduced during residency and may result in burnout. Interventions prioritized by residents can be grouped according to these four factors: **autonomy** (e.g., flexible schedule, forum to influence change), **meaning** (e.g., sensible patient census, support staff to offload clerical burdens), **competence** (e.g., protected learning time, feedback from faculty), and **relatedness** (e.g., social support).

The literature has not clarified guidance on NGT sample size, and this study, like others before, used a small number of focus groups. We were aware that the restriction of our project timeframe to conduct focus groups may have led to limited depth of discussion of each attribute. Subsequent groups may have been able to broadened the scope and breadth. We used eight systematic literature reviews of interventions, both individual and institutional, for prevention of burnout, to develop the list of 23 attributes. In addition, we spoke with experts to refine our list. However, we cannot rule out other burnout program attributes that are important to some residents or that additional groups of residents may have added other attributes not mentioned in the current study methods. In addition, the samples for the focus groups contained a relatively high proportion of internal medicine and family medicine residents but a limited representation of several other specialties. Participants also represented three diverse healthcare centers. Some might consider our selection of participants to be less generalizable to all residency programs and medical specialties. Future studies could do this procedure on each medical speciality and compare and contrast the program attribute rankings in interesting ways. A further limitation is that we have not compared the attributes derived from NGT with other approaches (e.g., Delphi, best-worst scaling, brainstorming). Head-to-head comparisons of different techniques could help to assess and understand strengths and weaknesses between approaches.

This study identified and ranked burnout prevention preferences in a resident population, showing that there is a need to address multiple organizational factors leading to burnout. There is also a need for complex interventions that will target several systemic and program level factors. While there was variability about attributes the groups perceived to be less important, as well as variability between healthcare centers, there was strong agreement about the most highly rated attributes. The attributes group naturally into themes of autonomy, meaning, competency, and relatedness. The next steps in this process will enable the researchers to refine the methods to determine measures for each attribute. The results of this study may help residency program directors understand what specific attributes of a burnout prevention program residents will value. The results will also be used to design a DCE study that would model residents’ burnout prevention program preferences. This may advance understanding and support for evidence-based, best practices as well as increase residents participation in, and adoption of, burnout prevention programs resulting in a potential decrease in rates of resident burnout. These same attributes could also be explored with physicians across specialities to determine whether the four themes of autonomy, meaning, competency, and relatedness also apply after board certification and across specialties.

## Supporting information

S1 Annex (DOCX)
