# Supplementary material for: Using nominal group technique among resident physicians to identify key attributes of a burnout prevention program
Source: PLoS One. 2022 Mar 18;17(3):e0264921. doi: 10.1371/journal.pone.0264921 (PMC8932600; doi:10.1371/journal.pone.0264921)
Supplement: S1 Annex — (DOCX) [file pone.0264921.s001.docx]

**Annex**

**Keywords:** We conducted our search for review studies and systematic reviews on interventions for physician burnout using the following keywords and databases

| **Database** | **Keyword** | | |
| --- | --- | --- | --- |
| PubMed | Review | In combination with | Physician |
| Embase | Systematic review |  | Resident |
| Web of Science | Burnout |  | Medical student |
| Cochrane | Intervention |  | Intern |
| CINAHL | Prevention |  | Medical residency |
| PsycINFO | Reduce burnout |  | Medical trainees |
| ERIC | Stress |  | Medical education |
| Academic Search Complete | Resiliency |  |  |

Inclusion criteria: a) study type: review studies and systematic reviews that included research on individual and organization-directed interventions to reduce burnout; b) the range of results: review studies and systematic reviews in which burnout, its consequences, or at least one of its main symptoms (emotional exhaustion, depersonalization) has been measured; c) study population: review studies and systematic reviews that studied medical students, residents, and physicians.

Exclusion criteria: a) review research with no predefined criteria to assess the quality of methodology; b) narrative and theoretical reviews; c) review studies and systematic reviews in which the research population are not predominantly physicians, medical students, and/or residents.
